# A Review of the Usage and Post-surgical Outcomes of Anesthesia for Laser In Situ Keratomileusis (LASIK) Procedure

**DOI:** 10.7759/cureus.30241

**Published:** 2022-10-12

**Authors:** Parsa Riazi Esfahani, Akshay J Reddy, Dillon A Sommer, Muhammad S Ghauri, Pasha Mazboudi, Monica E Guirgus, Danny S Abdalnour, Casey A Curow, Neel Nawathey, Mark Bachir, Hetal Brahmbhatt

**Affiliations:** 1 Medicine, California University of Science and Medicine, Colton, USA; 2 Neurosurgery, California University of Science and Medicine, Colton, USA; 3 Health Sciences, California Northstate University, Rancho Cordova, USA; 4 Psychiatry, Mercy General Hospital, Sacramento, USA

**Keywords:** dosage, oxybuprocaine, proparacaine, anesthesia, lasik

## Abstract

When laser in situ keratomileusis (LASIK) surgery is employed for myopia, hyperopia, and astigmatism, the process requires the usage of anesthetics to ensure that there is minimal patient harm and negative consequences once the procedure is complete. Statistical analysis was conducted as part of this review to evaluate the application of and distinctions between the different analgesics used for LASIK surgery by compiling and filtering information from multiple research studies. Topically administered oxybuprocaine and proparacaine were found to be the most commonly used anesthetics for LASIK, according to the data included in the review. It was also determined that there were no significant differences in terms of patient outcomes and drug concentrations when proparacaine was substituted for oxybuprocaine. This is particularly intriguing given their different chemical compositions. Temporary dry eyes were the most commonly reported adverse effect of LASIK when the anesthetic was employed. Perhaps cocaine derivatives produce similar anesthetic and post-surgical effects, but further investigations are needed to verify this hypothesis.

## Introduction and background

Laser in situ keratomileusis (LASIK) surgery is among the most frequently performed procedures worldwide. Nearly 60% of adults in the United States are affected by a refractory error, which explains why approximately one million individuals undergo corneal refractive surgery annually [1]. According to a meta-analysis of the US Food and Drug Administration (FDA)-approved LASIK device studies, 97% of patients achieved uncorrected visual acuity (UCVA) of 20/40, and 62% of patients achieved UCVA of 20/20. Since its inception 30 years ago, LASIK has continued to provide safe, efficient, and predictable results, with patients reporting greater satisfaction with the procedure compared to using traditional glasses or contact lenses [2]. LASIK alters the cornea’s refractive power in myopic, hyperopic, and astigmatic patients. This is achieved by utilizing a laser to create a hinged corneal flap from the epithelium, Bowman’s membrane, and the superficial part of the corneal stroma [3]. Before surgery, various parameters (i.e., thickness, refractive index, etc.) of the cornea are evaluated by keratometry and pachymetry. Topography and tomography are crucial for good refractive screening and have become the standard of care for preoperative screening [4].

Since patients are mostly responsive during these elective procedures, achieving effective intraoperative and postoperative pain control is paramount [5]. Currently, the standard of care in terms of anesthesia for LASIK varies as there is no singular anesthetic that is universally used for every LASIK procedure. Conventional regional anesthetics include eye drops that inhibit pain signals to the ocular nerve. In some cases, concomitant administration of topical anesthesia has improved the quality of care for patients. Overall, the choice of anesthetic is largely based on preoperative factors (i.e., comorbidities, surgeon’s preference, availability) that may influence patient safety, comfort, and outcomes [6]. To date, few studies have effectively compared the use of anesthetics historically used in LASIK surgery. In light of this, this review aimed to elucidate the benefits and outcomes of various anesthetics administered during ocular surgery.

## Review

Methods

**Figure 1:**
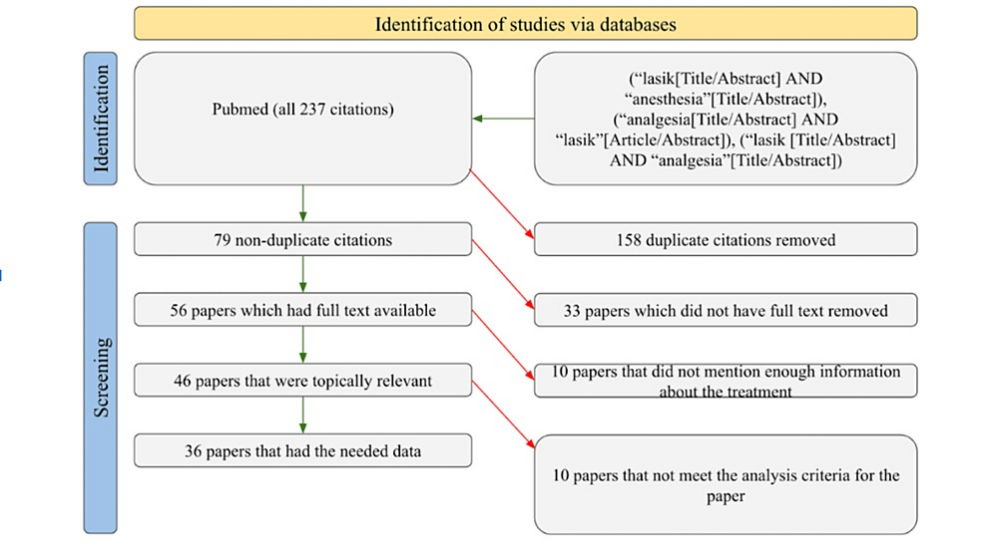
PRISMA diagram depicting the study selection process PRISMA: Preferred Reporting Items for Systematic reviews and Meta-Analyses

We conducted a search on PubMed to find studies about the use of anesthesia in LASIK surgery. Exact searches were done with the keywords “lasik and anesthesia,” “analgesia and lasik” and “lasik surgery.” We placed no restrictions in terms of time frames in the search. The search elicited a total of 237 studies, of which 79 were not duplicates, 56 had full text available, 46 were topically relevant studies, and only 36 had the relevant information needed and fulfilled the analysis criteria of our review. The data collected from these 36 studies included the type of anesthetic that was used, the dosage of the anesthetics that were used, the area of anesthetic application, and relevant information about post-treatment patient outcomes. Studies that did not provide sufficient information about at least two of these categories were excluded. This was done to avoid personal bias. Figure 1 provides a clear illustration of the filtering procedure used by the authors of this review.

LASIK surgery

In the normal eye, the cornea curves in a way that allows light to get refracted directly onto the retina. However, in patients with imperfect lens curvature (astigmatism), farsightedness (hyperopia), and nearsightedness (myopia), light is refracted incorrectly resulting in refractive errors [7]. Refractive surgery is any type of procedure that minimizes or corrects refractive errors. LASIK is the most commonly performed as well as the best-known type of refractive surgery [8]. During this procedure, the front of the eye, specifically the cornea, which is the dome-shaped clear tissue, is changed using a cutting laser to improve vision. Typically the anesthesia used in this procedure is in the form of eye drops that inhibit pain signals to the ocular nerve [9]. Thus patients are mostly awake during this procedure. This procedure is used for myopia, hyperopia as well as astigmatism in order to correct refractive errors. Including regional as well as topical anesthesia in ocular surgeries has improved the quality of care for patients [9]. The choice of anesthetic should be based on factors that may influence safety, comfort, cooperation as well as the comorbidities of the patient. Additionally, there are challenges associated with securing an airway in cases where there is limited access to the head of the patient. In this paper, we will be exploring how anesthetics are used specifically for LASIK procedures.

According to the data reported in Table 1, similar to many eye surgeries, LASIK is most typically performed under topical anesthesia in the form of eye drops [10-45]. Presumably, eye drops remain a preferred route of anesthesia administration due to their localized numbing effects on only the eye. Local anesthetics are generally safer and associated with fewer side effects than other types of anesthesia [9]. A patient under a local anesthetic remains conscious and does not require ventilatory support. The duration of nerve blocking in a local anesthetic is also not long; as a result, the recovery time for patients who are administered local anesthetics is shorter. Thus, pain control via topical anesthesia has proven to be especially useful in short procedures such as LASIK [9]. However, it is important to note that topical anesthesia may not provide complete anesthesia due to insufficient blocking of the ocular nerves. Subsequently, patients may move uncontrollably due to discomfort and pain, or even surgical anxiety. This can ultimately result in unexpected outcomes during a LASIK procedure, thereby leading to poor patient outcomes [9]. It should also be noted that the pain experienced can vary from patient to patient and it can be exacerbated by pre-existing conditions that patients may have. Collectively, it can be recognized that using a sufficient dosage of a topical anesthetic is highly critical for high patient satisfaction among patients who undergo LASIK surgery.

**Table 1:** The usage and application of anesthesia during LASIK surgery LASIK: laser in situ keratomileusis

Author (year)	Primary anesthetic used	Dosage	Area of application	Post-surgery complications
Abdelkader et al. (2006) [10]	Ketamine hydrochloride	50 mg/kg	Intramuscular	Ectasia
Asensio et al. (2003) [11]	Oxybuprocaine	0.4%	Topical	Changes to corneal thickness
Buratto and Ferrari (1997) [12]	Oxybuprocaine	4%	Topical	Hyper/hypo correction
Demirok et al. (2013) [13]	Proparacaine	0.5%	Topical	Dry eye
Donnenfeld et al. (2003) [14]	Proparacaine	1%	Topical	Dry eye
Donnenfeld et al. (2004) [15]	Proparacaine	1%	Topical	Loss of corneal sensation
Edelhauser (2000) [16]	Lidocaine	1%	Intraocular	N/A
Elder et al. (2000) [17]	Lignocaine	4%	Topical	N/A
Gunton and Armstrong (2010) [18]	N/A	N/A	Topical	Diplopia
Konomi et al. (2008) [19]	Oxybuprocaine	0.4%	N/A	Dry eye
Lam et al. (2012) [20]	Proparacaine	0.5%	Topical	N/A
Leaming (2003) [21]	Lidocaine	N/A	Topical	N/A
Leaming (2004) [22]	Lidocaine	N/A	Topical	N/A
Li et al. (2013) [23]	N/A	N/A	Topical	Dry eye
Magli et al. (2009) [24]	Oxybuprocaine	2%	Topical	Esotropia
Mahfouz and Khalaf (2005) [25]	Propofol/fentanyl (P/F) and ketamine/midazolam (K/M)	N/A	Topical	N/A
Moshirfar et al. (2014) [26]	Tetracaine and proparacaine	0.5% both	Topical	N/A
Nucci and Drack (2001) [27]	Oxybuprocaine or lidocaine	2%	Topical	Uveitis
Ortak et al. (2013) [28]	Proparacaine	0.5%	Topical	Thinner cornea
Oshika et al. (1999) [29]	N/A	N/A	Topical	Posterior capsular rupture
Oshika et al. (2000) [30]	N/A	N/A	Topical	Posterior capsular rupture
Oshika et al. (2001) [31]	N/A	N/A	Topical	Displacement of the vitreous
Patel et al. (2008) [32]	Bupivacaine	10%	Topical	None
Paysse et al. (2003) [33]	Halothane/nitrous oxide	N/A	Topical	None
Primack et al. (2001) [34]	Sevoflurane	N/A	Topical	Monocular diplopia
Sachdev et al. (2002) [35]	N/A	N/A	Topical	Epithelial defects
Saed and Abdrabbo (2011) [36]	Ketamine	1.5–2 mg/kg	Intravenous	None
Solomon et al. (2003) [37]	N/A	N/A	Topical	Lamellar flap
Tanaka et al. (2004) [38]	Oxybuprocaine	0.4%	Topical	Dry eye
Terry and Ousley (2006) [39]	Proparacaine	N/A	Topical	Disk dislocation
Toda et al. (2001) [40]	Oxybuprocaine	0.4%	N/A	Dry eye
Toda et al. (2002) [41]	Oxybuprocaine	0.4%	N/A	Dry eye
Wang et al. (2014) [42]	Proparacaine	0.5%	Topical	Respiratory depression, hypoxemia, apnea
Xu and Yang (2014) [43]	Ofloxacin	0.3%	N/A	Dry eye
Yin et al. (2007) [44]	Oxybuprocaine	0.4%	Topical	N/A
Yoon et al. (2009) [45]	N/A	N/A	Topical	Dry eye

Proparacaine and oxybuprocaine

According to the data presented in Table 1, it was identified that both proparacaine and oxybuprocaine were used most consistently across LASIK procedures [10-45]. Proparacaine was used commonly at a dosage of 0.5% or 1%. Oxybuprocaine was used mostly at a dosage of either 0.4% or 2% across different LASIK procedures. Both anesthetics are used most frequently across LASIK procedures due to their effectiveness as local, topical anesthetics. What was especially interesting about these two anesthetics was that, according to the data presented in Table 2, the concentration of oxybuprocaine and proparacaine that was used for LASIK was not proven to be statistically different. Although these two anesthetics may differ in some functional properties, they are both cocaine derivatives, and hence it makes sense that they would produce similar anesthetic effects at similar concentrations [20,41-42]. The data presented in Table 2 also established that the incidence rate of dry eye post-LASIK was not significantly different when oxybuprocaine was used instead of proparacaine. Furthermore, these anesthetics avoid extreme complications or side effects. One of the only recurring side effects was dry eyes; however, no severe complications were reported. Considering the wide range of complications that might arise from anesthesia, both proparacaine and oxybuprocaine have proven to be efficient, safe anesthetics during LASIK procedures, and they help to avoid further complications post-procedure [13,14,38-42]. The use of these topical anesthetics also provides physicians with a safe and accurate method of application. Physicians can steadily and accurately apply these medications in specific regions of the eye to obtain optimal results and avoid further problems [10,14-15]. An example of such an application may involve placing a drop of proparacaine or oxybuprocaine on the superior and inferior conjunctiva as opposed to the cornea to reduce incidences of documented side effects [20]. Furthermore, due to the reliability and accuracy of these topical anesthetics, physicians can increase the effect by having patients close their eyelids in order to spread the medication across the surface area of the eye. These methods allow for easy and efficient application and spread, minimizing the risks associated with LASIK and anesthesia.

**Table 2:** Statistical significance between group means

Group comparison	t value	P-value
Concentration of oxybuprocaine vs. proparacaine	0.4017	0.694
Side effects of dry eye in oxybuprocaine vs. proparacaine	0.8033	0.4343

Surgical complications

Based on the information provided in Table 1, post-surgical complications of the severe kind were rarely observed in the majority of patients following LASIK [10-46]. Blindness is an example of a complication that can occur; however, the incidence rate of this event is extremely low [10-20]. Most notably, numerous patients reported dry eyes post-surgery. Dry eye syndrome (DES) is typically transient in nature, lasting 6-12 months on average, with symptoms peaking within the first several months [13]. DES pathophysiology is not fully understood and is believed to have a multifactorial and complex basis [14-15]. However, a commonly accepted theory for the etiology of dry eyes post-LASIK involves the iatrogenic damage to the dense subbasal nerve plexus and stromal corneal nerves that occurs during the creation of the anterior corneal flap [19,23]. This incision and the proceeding use of suction on the LASIK flap likely causes additional damage to and loss of conjunctival goblet cells. These cells secrete soluble mucins to the tear film that protects and lubricates the ocular surface, the subsequent loss of which can lead to DES. Other mechanisms for DES such as limited corneal sensation and the blink reflex, reduced tear-film stability, post-surgical centralized inflammation, and the use of topical anesthetics prior to corneal flap creation have all been associated with post-LASIK DES [40-41]. While DES is increasingly common among post-LASIK patients, it has been noted that the prevalence of some dry eye symptoms prior to LASIK is estimated to be between 38 and 75% [46]. This is likely due to contact lens intolerance in those patients and skews the population of those with DES post-LASIK and should be kept in mind when analyzing data.

In this study, we were able to identify several different anesthetics that have been historically used for the LASIK procedure [10-45]. By comparing the observed outcomes and identifying the associated risks with the different anesthetics in the studies described, physicians will have a wider variety of anesthetics to choose from to meet the needs of the patient and thus provide more successful outcomes. Additionally, we are hopeful that with the information collected from the various studies regarding the effectiveness of varying dosages, physicians will have a basis to effectively alter the dosage of anesthesia to mediate risks of adverse side effects while also retaining the efficacy of the drug. Moreover, we sought to highlight different areas of application for physicians to identify their options and use a data-driven approach in selecting the area of application with the highest likelihood of efficacy. Furthermore, we consolidated the reported post-surgery complications from LASIK surgeries to help physicians with considerations, thereby allowing an effective way to compare and contrast data from LASIK procedures.

Future applications and current limitations

This review was able to effectively examine the effects of various means of applications and anesthetic dosages on LASIK procedures. Through an analysis of 36 studies, we were able to specifically detect the effects of different dosages and applications of anesthetics on post-surgical complications [10-45]. Some observations pertained to studies that applied anesthetics topically, a majority of which had post-surgical complications of dry eye. One of the limitations of this review was that a majority of the analyzed studies utilized topical anesthetic rather than intramuscular, intravenous, or intraocular variants. Therefore, future studies should compare various means of application of anesthetics equivalently. For example, a future study may look at 10 studies that applied anesthetics topically, 10 that applied anesthetics intramuscularly, 10 that applied anesthetics intravenously, and 10 that applied anesthetics intraocularly. This would allow for a clear insight into whether or not a post-surgical complication of dry eye is more prevalent among topical anesthetics relative to other applications. A second limitation of this study was the limited variety of anesthetics observed. There were less than 20 anesthetics analyzed. We recommend that future studies should aim to analyze at least 30 anesthetics or more in order to determine if the trends found in this study are statistically significant. With these considerations in mind, the effects of anesthetics on LASIK surgery should be explored further.

Despite the existing studies on the administration and use of proparacaine and oxybuprocaine in LASIK patients, additional research is required to identify more appropriate standards that may be used to prevent adverse effects during these operations. Specifically, further research is required to determine the effects of proparacaine in conjunction with combination medicines. During such investigations, substances such as ethylenediaminetetraacetic acid (EDTA) may be necessary to determine the impact of various compounds on the analgesic properties of proparacaine [47]. Appropriate anesthetic administration is essential for decreasing the pain that LASIK patients may experience. Individuals with myopia, on the other hand, should be recognized early to minimize unfavorable health implications and diminishing surgical results. Future development of artificial intelligence (AI) software that analyzes retinal scans could help with identifying myopic people. To ensure the safety of LASIK patients, additional research on proparacaine's side effects is required. Future research could possibly focus on finding a solution to eliminate the requirement for LASIK surgery. A change in lifestyle during pregnancy or even early infancy could potentially decrease the development and progression of myopia, as has been shown with diseases such as Alzheimer's. Regardless of the circumstances, additional research into myopia and LASIK is necessary to enhance patient outcomes.

## Conclusions

The authors compiled and filtered through several studies to produce a review article that discussed information about the application of and differences between the various types of anesthetics used for LASIK surgery. When anesthesia was used in an appropriate and effective manner, patient outcomes were generally favorable. The most commonly reported negative consequence of LASIK when anesthesia was utilized was dry eyes for a brief period of time. According to the data presented in the review, the most commonly utilized anesthetics for LASIK were oxybuprocaine and proparacaine, which were applied topically. What was particularly interesting was that after the proper statistical analysis was conducted, we found no significant differences in patient outcomes and drug concentrations when proparacaine was used in place of oxybuprocaine, which is interesting given that they have different chemical compositions. Perhaps future studies should verify whether or not patient outcomes post-surgery would be statistically different if different cocaine derivatives were used as anesthetic agents.
